# Lung-protective ventilation increases cerebral metabolism and non-inflammatory brain injury in porcine experimental sepsis

**DOI:** 10.1186/s12868-021-00629-0

**Published:** 2021-04-29

**Authors:** Axel Nyberg, Erik Gremo, Jonas Blixt, Jesper Sperber, Anders Larsson, Miklós Lipcsey, Andreas Pikwer, Markus Castegren

**Affiliations:** 1 Centre for Clinical Research Sörmland, Uppsala University, Uppsala, Sweden; 2 Department of Medical Sciences, Uppsala University, Uppsala, Sweden; 3 Perioperative Medicine and Intensive Care (PMI), Karolinska University Hospital, Stockholm, Sweden; 4 The Department of Physiology and Pharmacology (FyFa), Karolinska Institute, Stockholm, Sweden; 5 Hedenstierna Laboratory, CIRRUS, Anesthesiology and Intensive Care, Department of Surgical Sciences, Uppsala University, Uppsala, Sweden; 6 Department of Anaesthesiology & Intensive Care, Centre for Clinical Research, Sörmland, Mälarsjukhuset, 631 88 Eskilstuna, Sweden

**Keywords:** Innate immunity, Biomarkers, Microdialysis, Tidal volume, Endotoxin

## Abstract

**Background:**

Protective ventilation with lower tidal volumes reduces systemic and organ-specific inflammation. In sepsis-induced encephalopathy or acute brain injury the use of protective ventilation has not been widely investigated (experimentally or clinically). We hypothesized that protective ventilation would attenuate cerebral inflammation in a porcine endotoxemic sepsis model. The aim of the study was to study the effect of tidal volume on cerebral inflammatory response, cerebral metabolism and brain injury. Nine animals received protective mechanical ventilation with a tidal volume of 6 mL × kg^−1^ and nine animals were ventilated with a tidal volume of 10 mL × kg^−1^. During a 6-h experiment, the pigs received an endotoxin intravenous infusion of 0.25 µg × kg^−1^ × h^−1^. Systemic, superior sagittal sinus and jugular vein blood samples were analysed for inflammatory cytokines and S100B. Intracranial pressure, brain tissue oxygenation and brain microdialysis were sampled every hour.

**Results:**

No differences in systemic or sagittal sinus levels of TNF-α or IL-6 were seen between the groups. The low tidal volume group had increased cerebral blood flow (*p* < 0.001) and cerebral oxygen delivery (*p* < 0.001), lower cerebral vascular resistance (*p* < 0.05), higher cerebral metabolic rate (*p* < 0.05) along with higher cerebral glucose consumption (*p* < 0.05) and lactate production (*p* < 0.05). Moreover, low tidal volume ventilation increased the levels of glutamate (*p* < 0.01), glycerol (*p* < 0.05) and showed a trend towards higher lactate to pyruvate ratio (*p* = 0.08) in cerebral microdialysate as well as higher levels of S-100B (*p* < 0.05) in jugular venous plasma compared with medium–high tidal volume ventilation.

**Conclusions:**

Contrary to the hypothesis, protective ventilation did not affect inflammatory cytokines. The low tidal volume group had increased cerebral blood flow, cerebral oxygen delivery and cerebral metabolism together with increased levels of markers of brain injury compared with medium–high tidal volume ventilation.

**Supplementary Information:**

The online version contains supplementary material available at 10.1186/s12868-021-00629-0.

## Background

Mechanical ventilation (MV) carries the risks of cyclic atelectasis and overdistension of alveoli, events that add to a pro-inflammatory response [1]. Protective ventilation, i.e. the avoidance of iatrogenic harm by the use of higher than traditional positive end-expiratory pressure (PEEP) levels and smaller than traditional tidal volumes (V_T_), has an established role in the care of patients with acute respiratory distress syndrome (ARDS) [2]. Arguments favoring protective ventilation in patient categories other than those suffering from ARDS to prevent rather than to treat manifest lung injury have been raised [3]. Experimental large animal studies have shown strong associations between lower tidal volumes and decreased systemic and organ-specific inflammation, as well as decreased microbiological growth [4–6].

Sepsis-associated encephalopathy (SAE), defined as a transient and reversible brain dysfunction, occurs when the source of sepsis is located outside of the central nervous system. SAE affects nearly 30% of septic patients at admission and is a known risk factor for mortality [7]. The pathophysiology of SAE is highly complex and not fully understood, but proposed mechanisms include alterations in the blood–brain barrier, effects of local and systemic inflammatory cytokines, cerebral metabolism disruption and imbalance of neurotransmitters [8, 9].

Protective ventilation, or more specifically, permissive hypercapnia carries the risk of elevated intracranial pressure and possibly compounding brain injury [10]. Although the use of protective ventilation in critically ill patients with primary neurosurgical and neurological conditions can be used without threatening cerebral perfusion, the safety of protective ventilation in patients with refractory raised intracranial pressure is not well established [11]. While protective ventilation has been associated with attenuation of systemic inflammation, less is known about the effects of protective ventilation on cerebral inflammation, brain metabolism and central nervous vascular physiology. The possible implication of protective mechanical ventilation strategy in acute brain injury patients has been described as an unexplored area of research in both experimental and clinical settings [12].

The current study hypothesized that protective ventilation would attenuate the cerebral inflammation as compared with ventilation with medium–high tidal volumes. The primary aim was to study the effect of two tidal volumes on cerebral plasma concentrations for two inflammatory cytokines, tumour necrosis factor alpha (TNF-α) and interleukin 6 (IL-6). A secondary goal was to study whether protective ventilation would affect the cerebral metabolism and markers of brain injury.

## Results

The animals weighed 28.2 kg (± 2.8) and in good health when they reached the research facility. All animals survived until the experimental endpoint. Three animals in the Medium high V_T_ group and two animals in the Low V_T_ group received noradrenalin during the experiment according to the protocol. One animal in each group received a fluid bolus after baseline according to the protocol.

The physiologic parameters in groups Medium high V_T_ and Low V_T_ are listed in Table 1. Tidal volumes and respiratory rate were, according to the protocol, significantly different between the groups. Peak airway pressures in group Low V_T_ were slightly lower than in group Medium high V_T_. Static pulmonary compliance was nearly significantly lower in group Low V_T_. Arterial blood gases were kept within the pre-set limits and did not differ between the groups.

**Table 1:** Physiologic data during the experiment

	Group	0	1	2	3	4	5	6	*p*
TVe	Low V_T_	163 ± 16	163 ± 15	163 ± 15	164 ± 15	166 ± 11	165 ± 12	165 ± 11	0.001
(cmH_2_0)	Medium high V_T_	244 ± 35	247 ± 37	245 ± 37	248 ± 38	247 ± 37	247 ± 37	247 ± 38	
RR	Low V_T_	46 ± 3	46 ± 4	48 ± 5	48 ± 5	48 ± 5	48 ± 5	48 ± 5	0.001
(min^-1^)	Medium high V_T_	24 ± 5	24 ± 5	24 ± 5	24 ± 5	24 ± 5	24 ± 5	24 ± 5	
P Peak	Low V_T_	19 ± 2	20 ± 2	20 ± 2	21 ± 1	21 ± 2	21 ± 2	21 ± 2	0.04
(cmH_2_0)	Medium high V_T_	21 ± 1	22 ± 1	22 ± 2	22 ± 2	22 ± 2	22 ± 2	23 ± 2	
Compliance	Low V_T_	23 ± 8	24 ± 8	22 ± 5	21 ± 6	21 ± 5	21 ± 4	19 ± 3	0.06
(mL x cmH_2_0^-1^)	Medium high V_T_	30 ± 6	27 ± 8	26 ± 7	26 ± 6	26 ± 5	25 ± 4	24 ± 5	
PaO_**2**_	Low V_T_	19.0 ± 1.2	15.8 ± 3.1	17.1 ± 2.2	15.3 ± 3.3	15.1 ± 2.9	15.4 ± 2.6	15.1 ± 2.9	0.16
(kPa)	Medium high V_T_	19.3 ± 1.5	17.8 ± 2.8	16.5 ± 3.8	16.5 ± 3.8	16.0 ± 3.8	17.7 ± 2.7	17.0 ± 2.3	
PaCO_**2**_	Low V_T_	5.6 ± 0.4	6.2 ± 0.7	5.6 ± 0.3	5.6 ± 0.4	5.5 ± 0.6	5.3 ± 0.5	5.3 ± 0.5	0.15
(kPa)	Medium high V_T_	5.1 ± 0.3	5.6 ± 0.5	5.5 ± 0.5	5.5 ± 0.5	5.3 ± 0.3	5.3 ± 0.6	5.2 ± 0.3	
PaO_**2**_/FiO_**2**_	Low V_T_	475 ± 31	395 ± 80	427 ± 55	381 ± 82	373 ± 80	380 ± 74	348 ± 78	0.66
(mmHg)	Medium high V_T_	484 ± 38	421 ± 120	404 ± 80	385 ± 76	405 ± 90	410 ± 75	379 ± 89	
Temp	Low V_T_	38.1 ± 0.4	38.3 ± 0.6	38.5 ± 0.8	38.8 ± 0.9	38.8 ± 0.9	38.7 ± 1.1	38.7 ± 1.1	0.29
(°)	Medium high V_T_	38.3 ± 0.8	38.8 ± 1.2	39.2 ± 1.4	39.4 ± 1.6	39.5 ± 1.9	39.5 ± 1.9	39.4 ± 2.1	
HR	Low V_T_	95 ± 25	106 ± 21	102 ± 15	111 ± 24	104 ± 18	104 ± 17	105 ± 16	0.45
(min^-1^)	Medium high V_T_	97 ± 17	110 ± 26	111 ± 21	108 ± 18	110 ± 21	111 ± 21	125 ± 29	
MAP	Low V_T_	83 ± 13	83 ± 19	96 ± 13	95 ± 20	92 ± 18	84 ± 19	83 ± 19	0.69
(mmHg)	Medium high V_T_	89 ± 11	95 ± 18	98 ± 17	98 ± 13	91 ± 19	83 ± 16	77 ± 15	
MPAP	Low V_T_	18 ± 3	37 ± 8	31 ± 5	35 ± 9	30 ± 7	28 ± 7	27 ± 7	0.15
(mmHg)	Medium high V_T_	19 ± 3	33 ± 9	30 ± 6	29 ± 6	25 ± 4	23 ± 4	24 ± 3	
ICP	Low V_T_	12 ± 6	17 ± 6	18 ± 7	21 ± 10	23 ± 9	25 ± 13	26 ± 15	0.72
(mmHg)	Medium high V_T_	17 ± 12	17 ± 7	19 ± 8	18 ± 6	17 ± 4	16 ± 4	18 ± 5	
CI	Low V_T_	3.4 ± 1.2	3.5 ± 1.2	3.2 ± 0.8	2.9 ± 0.9	2.9 ± 0.9	3.1 ± 0.7	3.2 ± 0.8	0.24
(L x min^-1^ x m^-2^)	Medium high V_T_	3.4 ± 0.5	2.8 ± 0.7	2.6 ± 0.5	2.5 ± 0.6	2.6 ± 0.5	2.8 ± 0.7	2.8 ± 0.6	
Lactate	Low V_T_	1.6 ± 0.6	1.5 ± 0.8	1.1 ± 0.5	1.2 ± 0.6	1.3 ± 0.6	1.1 ± 0.4	0.9 ± 0.3	0.95
(mmol x L^-1^)	Medium high V_T_	1.4 ± 0.3	1.8 ± 1.3	1.3 ± 0.3	1.3 ± 0.5	1.0 ± 0.3	0.9 ± 0.2	0.9 ± 0.2	
Diuresis	Low V_T_		40(25–50)	70(38–180)	90(55–170)	111(80–500)	175(60–340)	180(65–390)	0.71
(mL x h^-1^)	Medium high V_T_		95(50–160)	105(50–180)	160(80–270)	70(55–180)	90(40–290)	95(40–290)	

Values are mean ± SE or median (interquartile range). The p-values are results of general linear models with random effects. A *p *value of < 0.05 was considered significant

*TVe* expired tidal volume, *RR *respiratory rate, *P Peak *peak airway pressure, Compliance-Static airway compliance, *PaO*_2_ arterial partial pressure of oxygen, *PaCO*_2_ arterial partial pressure of carbon dioxide, *PaO*_2_*/FiO*_2_ arterial partial pressure of oxygen to fraction of inspired oxygen, *Temp* core body temperature, *HR* heart rate, *MAP* mean arterial pressure, *MPAP* mean pulmonary arterial pressure, *ICP* intracranial pressure, *CI* cardiac index

The biological response to the endotoxin infusion was evident in all animals with rising body temperature, decreased PaO_2_/FiO_2_ index, increased heart rate, MPAP and ICP. CI and arterial lactate decreased during the experiment. Even though the vital physiological parameters deteriorated during the experiment, no evident organ failure was noted. Apart from the differences in spirometric variables, no differences were observed between the groups. The results from the control animals are summarised in Additional file 1: Table S1.

### Cerebral blood flow and oxygen delivery

**Fig. 1:**
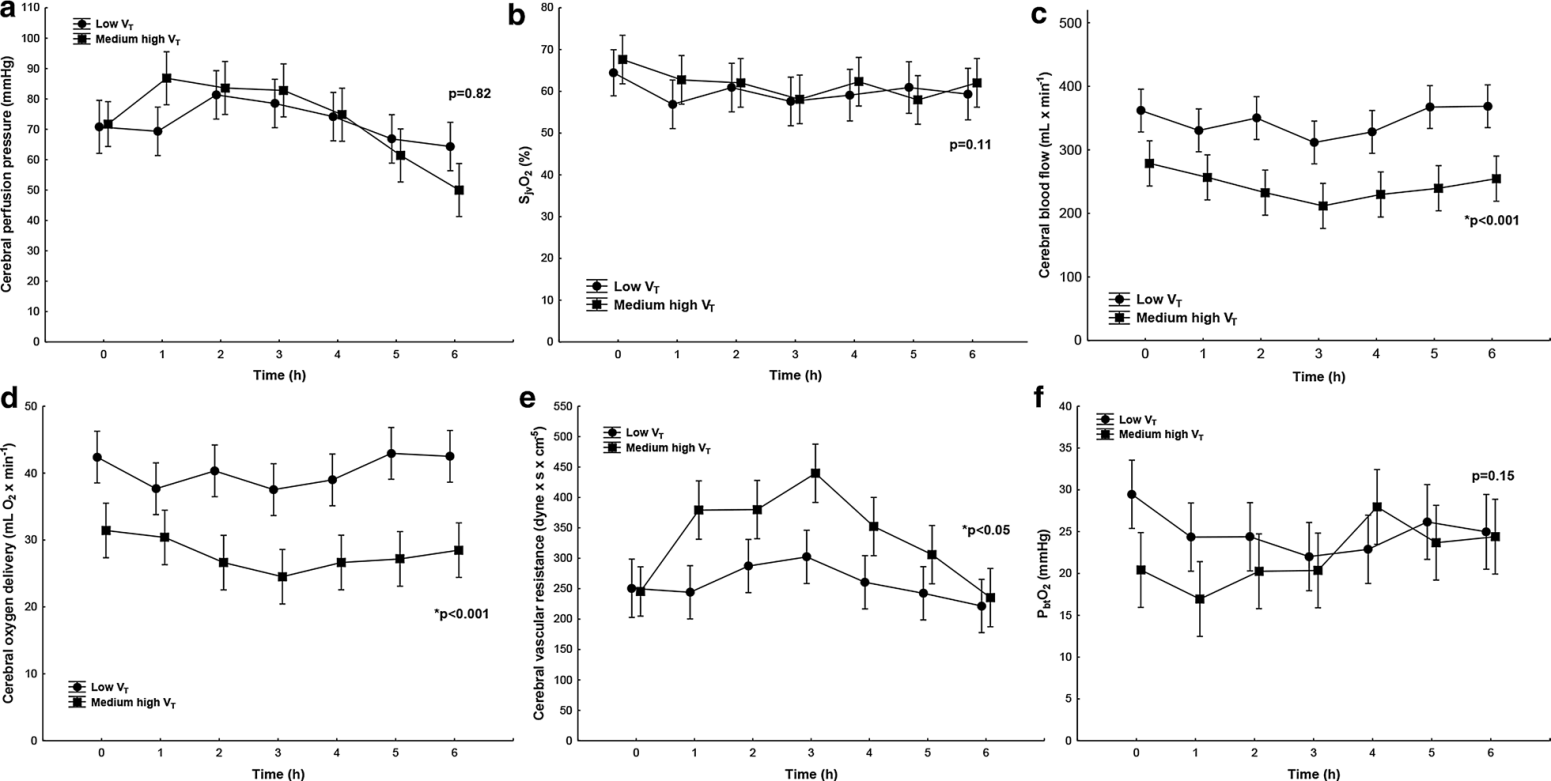
Cerebral circulation variables by groups. **a** Cerebral perfusion pressure. **b** Jugular vein oxygen saturation. **c** Cerebral blood flow. **d** Cerebral oxygen delivery. **e** Cerebral vascular resistance. **f** Partial pressure of oxygen in brain tissue. Values are mean ± SE. The *p* values are the results of general linear models with random effects. A *p* value of < 0.05 was considered significant and marked with *

Although no differences between groups were seen for cerebral perfusion pressure (CPP) and jugular bulb saturation (SjvO_2_) (Fig. 1a, b), cerebral blood flow and cerebral oxygen delivery were higher and the cerebral vascular resistance lower in group Low V_T_ compared with group Medium high V_T_ (Fig. 1c–e). There were no differences between groups in PtbO_2_ though group Low V_T_ had numerically higher values at the beginning of the experiment (Fig. 1f).

### Cerebral metabolism

**Fig. 2:**
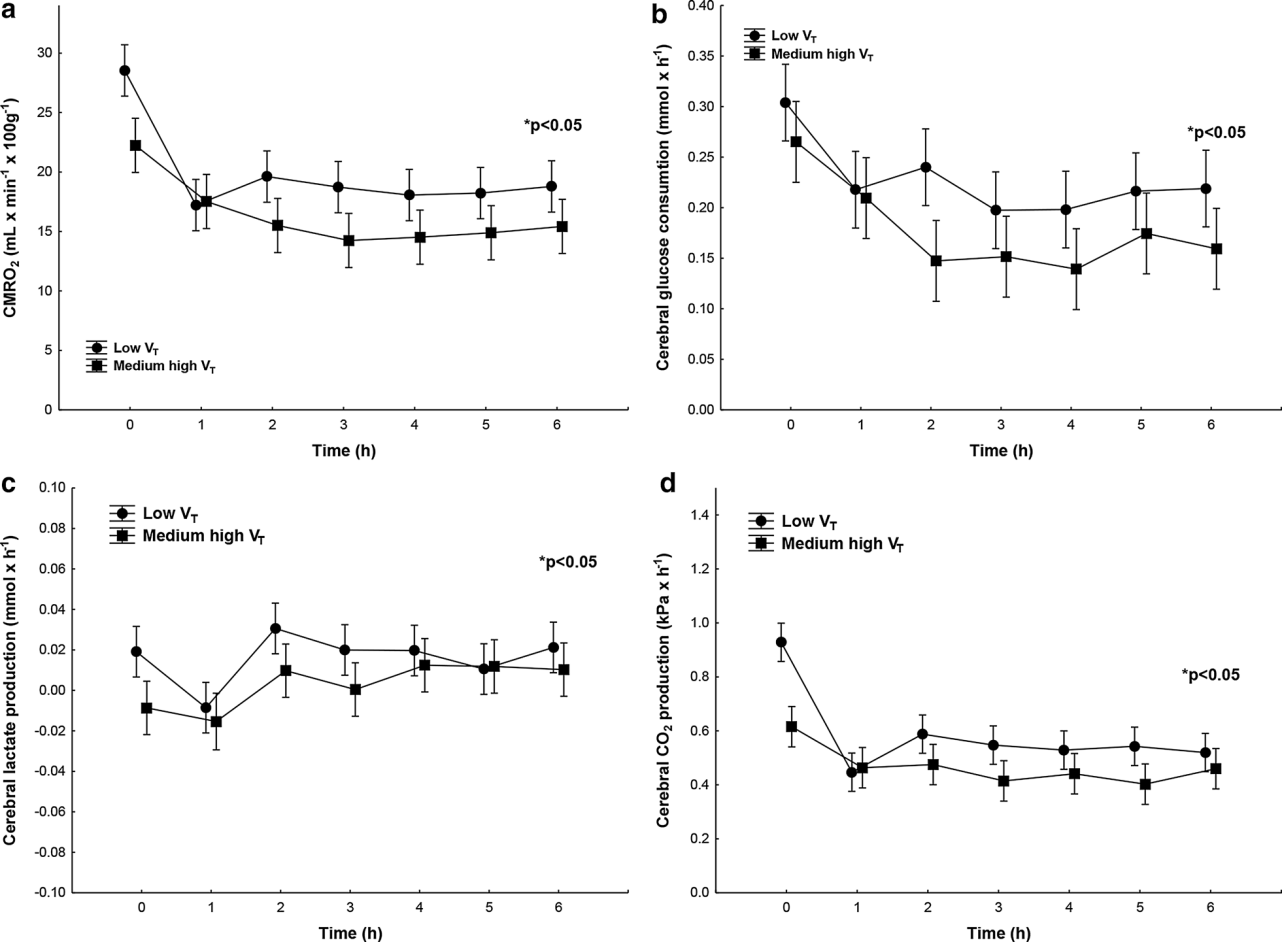
Cerebral metabolic variables by groups. **a** Cerebral metabolic rate of oxygen. **b** Cerebral glucose consumption. **c** Cerebral lactate production. **d** Cerebral carbon dioxide production. **e** Brain tissue partial oxygen pressure. Values are mean ± SE. The *p* values are the results of general linear models with random effects. A *p* value of < 0.05 was considered significant and marked with *

**Fig. 3:**
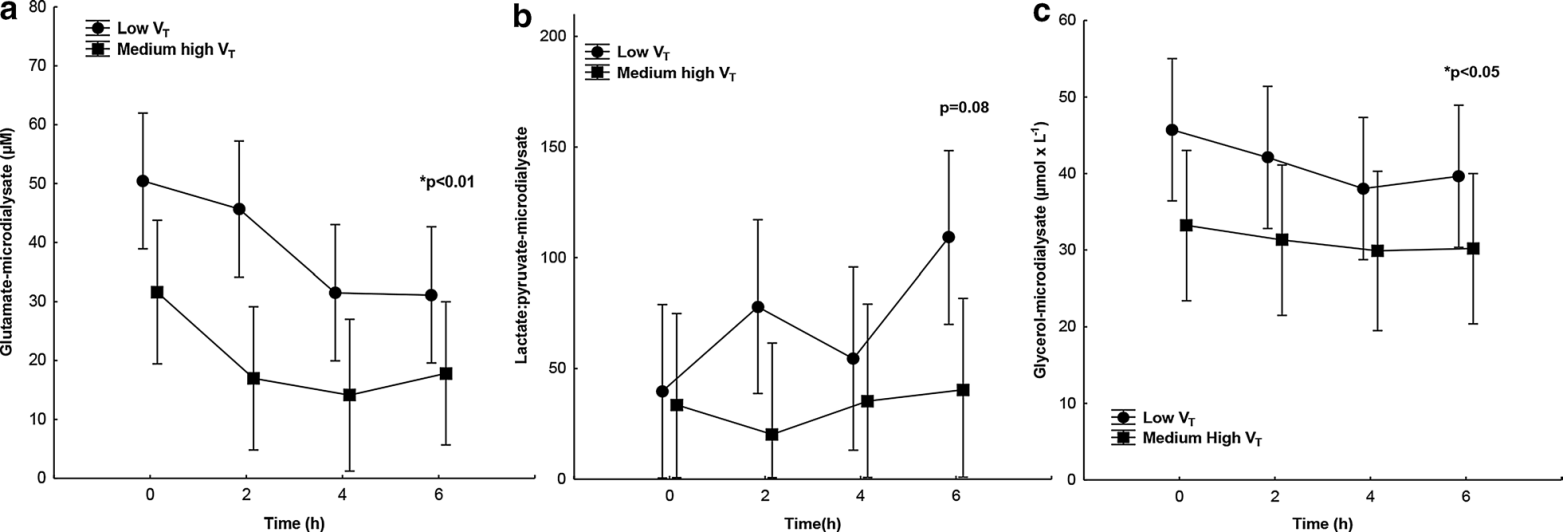
Microdialysate results. **a** Glutamate. **b** Lactate to pyruvate ratio c. Glycerol. Values are mean ± SE. The *p* values are the results of general linear models with random effects. A *p* value of < 0.05 was considered significant and marked with *

Cerebral metabolic rate of oxygen (CMRO_2_) decreased during the experiment but was higher in group Low V_T_ than in group Medium high V_T_ (Fig. 2a). The same pattern was seen as concerns cerebral glucose consumption and lactate and CO_2_ production (Fig. 2b–d). In brain tissue microdialysate glutamate decreased during the experiment but was higher in group Low V_T_ than in group Medium high V_T_ (Fig. 3a). The ratio of lactate to pyruvate in brain tissue microdialysate increased during the experiment and was highest in group Low V_T_ (Fig. 3b). Glycerol in microdialysate showed elevated levels during the experiment, being highest in group Low V_T_ (Fig. 3c).

### Biomarkers of inflammation and blood–brain barrier injury

**Fig. 4:**
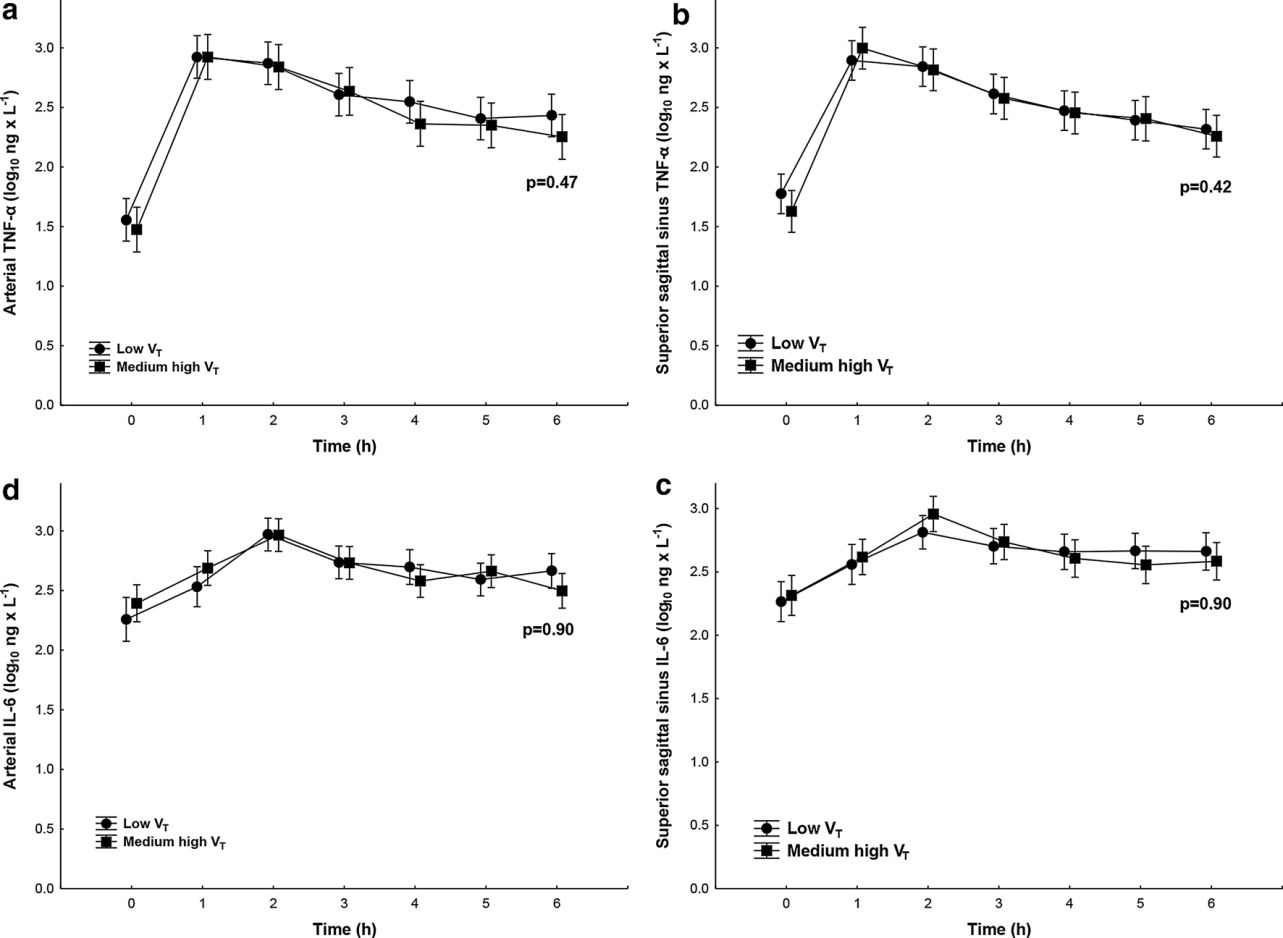
Plasma levels of inflammatory markers. **a** Arterial log_10_ TNF-α concentration. **b** Sagittal sinus log_10_ TNF-α concentration. **c** Arterial log_10_ IL-6 concentration. **d** Sagittal sinus log_10_ IL-6 concentration. Values are mean ± SE. The p-values are the results of general linear models with random effects. A *p* value of < 0.05 was considered significant and marked with *

**Fig. 5:**
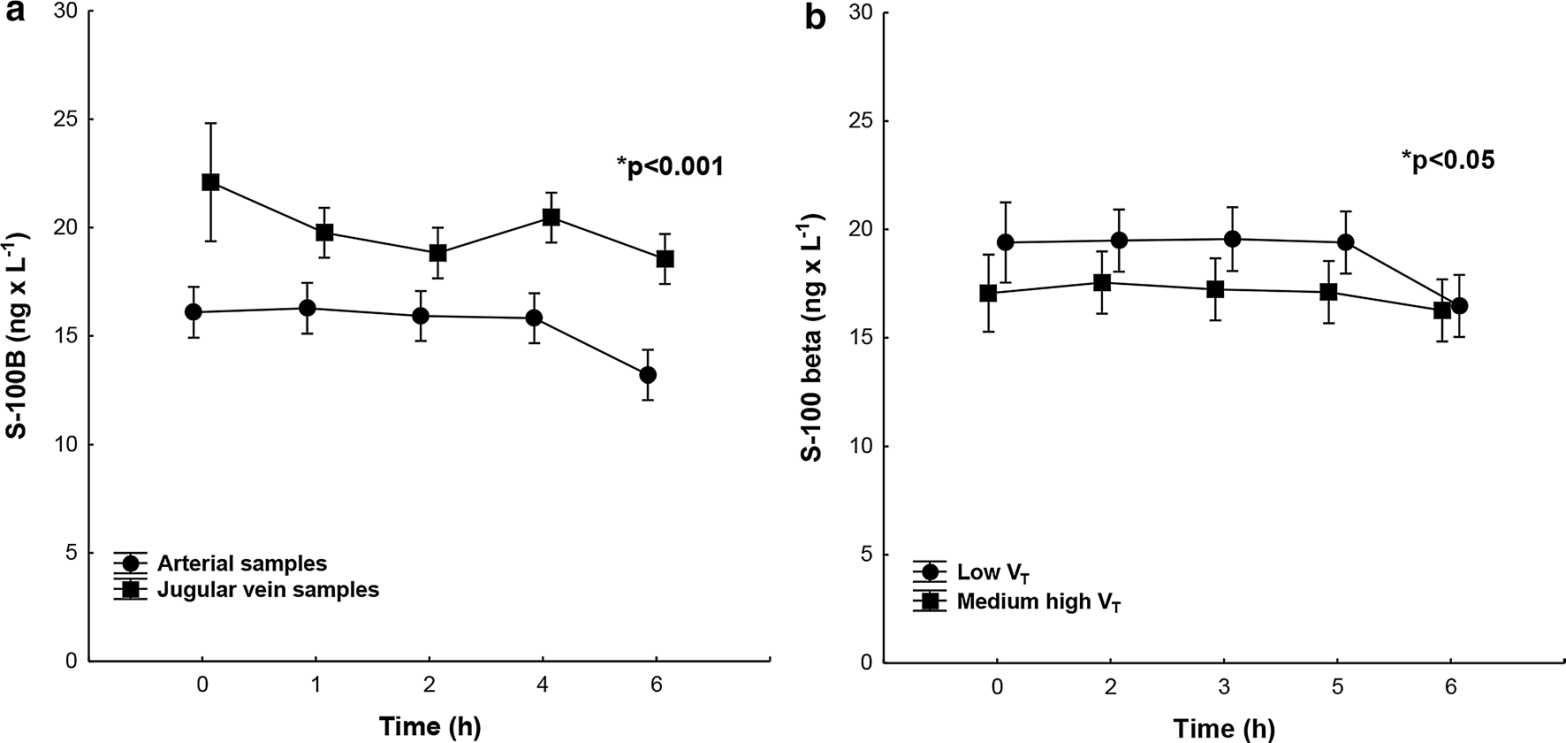
Plasma levels of S-100B. **a** S-100 B in the artery and the jugular vein. **b** S-100 in the two ventilation groups. Values are mean ± SE. The *p* values are the results of general linear models with random effects. A *p* value of < 0.05 was considered significant and marked with *

In both groups systemic inflammation was evident, with logarithmic increases in arterial and superior sagittal sinus plasma levels of TNF-α and IL-6. No differences between groups were observed (Fig. 4a–d). Plasma levels of S-100B were elevated during the experiment and higher in the jugular bulb than in the artery (Fig. 5a). Group Low V_T_ had higher jugular bulb plasma levels than group Medium high V_T_ (Fig. 5b).

## Discussion

The main finding of this study is that protective ventilation with lower tidal volumes is associated with increased cerebral perfusion and oxygen delivery as well as increased cerebral metabolism. These differences were not associated with differences in inflammatory cytokines. However, increased biomarkers of brain ischemia and injury in plasma and cerebral microdialysate were found in animals ventilated with lower tidal volumes.

Bickenbach et al. pursued the same research question as this study, also in pigs [13]. The setting of the study was acute lung injury and the authors compared the effects between tidal volumes of 6 mL × Kg^−1^ with a very harmful ventilator mode of 12 mL × kg^−1^ and extremely high peak airway pressures. The study noted lower systemic levels of inflammatory cytokines, S100B and increased cerebral metabolism following low tidal volume ventilation. Thus, in part, our study contrasts with the findings of Bickenbach et al. However, the group that was compared with the low tidal volume ventilation was ventilated with an obvious deleterious technique, which may have increased the possibility of detecting differences in inflammatory cytokines. In this study protective ventilation did not have any effect on systemic levels of the inflammatory cytokines. This result contrasts with earlier findings from our research group [4–6]. The design of this study was different from earlier studies, especially compared to postoperative experimental septic models. The animals in the present study were subjected to lower doses of endotoxin and the animals were not laparotomised before baseline. The lower systemic inflammatory stimulus in the present study was also evident in the absence of pulmonary, respiratory or renal organ dysfunction. A possible explanation of the absence of inflammatory differences between the groups might be the lower level of inflammation present in our study. The present study was deliberately designed to produce a state of systemic inflammation in the animals, although not to the level of multiorgan failure. The rationale for the design of the model was to enable the study of cerebral inflammation in the setting of general inflammation, with adverse effects of failing vital organs reduced to a minimum. The absence of an effect of different tidal volumes on cerebral inflammation refutes our main hypothesis. This absence, too, might be attributed to the lower level of systemic inflammation compared with previous investigations.

The study produced a clear picture of possibly injurious cerebral effects following ventilation with lower tidal volumes. Because the animals were ventilated with the same PEEP levels (the group with lower tidal volumes had lower peak airway pressures and no differences between groups were seen in arterial O_2_ or CO_2_ levels, systemic blood pressures or ICP), the data do not indicate that the reason for this difference was either macrorespiratory or circulatory.

The animals ventilated with a low V_T_ showed, quite unexpectedly, a combination of higher cerebral blood flow, lower cerebral vascular resistance, higher CMRO_2_, higher glucose consumption as well as both higher cerebral lactate and CO_2_ production. The low V_T_ animals also expressed higher levels of glutamate, glycerol, lactate to pyruvate ratio in brain microdialysis and higher plasma levels of S-100B. It could be speculated whether this combination of effects is part of the same mechanism or several concomitant mechanisms. In a study comparing different tidal volumes during experimental sepsis decreased systemic inflammatory cytokine levels were found in animals ventilated with lower tidal volumes. At the same time, however, the low tidal volume ventilation was associated with a profound and early endotoxin tolerance and higher levels of nitrite, possibly indicating induced iNOS activity [4]. The authors speculate that the higher respiratory rate or the higher respiratory flow rate in the lower tidal volume ventilation group that is needed to maintain normal carbon dioxide partial pressures in blood might have increased the activity of metalloproteinases in the lung and subsequent induction of iNOS, effects also described in rat experiments [14, 15]. Of note, in the study above, the animals ventilated with lower tidal volumes exhibited lower levels of jugular vein oxygen saturation, possibly indicating increased oxygen extraction and cerebral metabolism [4].

Pulmonary hypertension as an effect of endotoxin challenge is a key feature during porcine endotoxemia and has been shown to partly be associated with increased endothelin-1 levels in plasma [16]. Increased endothelin-1 levels in plasma have also been reported during human sepsis [17]. In the present study the animals ventilated with a low V_T_ had higher mean pulmonary arterial pressure and higher intracranial pressure, most notably towards the end of the experiment. Higher pulmonary arterial pressure during endotoxemia has been associated with plasma levels of endothelin-1 [18]. Both iNOS and endothelin-1 have been associated with astrocyte dysfunction in several neurological conditions, including brain ischemia [19, 20]. A recent study in rats further confirmed the association between tidal volume, endothelin-1 and NO synthetase [21]. Thus, one hypothesis to account for our results could be formulated as follows: low tidal volume ventilation, perhaps coupled with the effects of a higher respiratory rate, leads to increased endothelin levels and induced NO synthetase. Elevated levels of endothelin-1, metalloproteinase and NO levels could subsequently have exerted cerebral vasodilatory effects and concomitantly induced cerebral cellular injury, probably not ischemia related. Whether the cellular injury observed in the current study was caused by energy substrate depletion or direct cellular damage from metabolic or plasma components cannot be determined.

What this study adds to our previous knowledge is that even in a condition of low-level inflammation, without reaching a situation of acute lung injury or multiorgan failure, differences in tidal volume exert significant and possibly harmful effects on non-pulmonary organs. One could speculate whether the detrimental effects shown in this study would be outweighed by attenuated levels of inflammatory cytokines in the case of more prominent systemic inflammation and vital organ failure.

Our experiment was not designed to explain cerebral injury associated with low tidal volume ventilation using a mechanistic model. In the current situation there is no possibility to investigate endothelin-1 levels in plasma or tissues, NO levels or metalloproteinase activity in lungs or brain. Future research must pursue the hypothesis formulated above. Another limitation of our study is the short study period. However, because the primary aim was to study inflammatory and acute effects of tidal volume during endotoxemia, the long-term effects were not our aim. On the other hand, the study of large animals and the pig, in particular, gives the possibility to use an experimental setting and surveillance techniques remarkably similar to the human clinical setting. Thus, the translational value is higher than studies in rodents. The present results should stimulate research in both clinical and experimental contexts. Clinical studies of patients with neurocritical conditions in which multimodal neurointensive care is warranted could investigate whether lower tidal volumes, in comparison with higher tidal volumes, have effects on cerebral metabolism and brain injury in humans.

## Conclusions

Ventilation with lower tidal volumes (6 mL × kg^−1^) as compared with medium high tidal volumes (10 mL × kg^−1^) in porcine endotoxemia did not affect cerebral cytokine levels. However, low tidal volume ventilation increased cerebral perfusion, cerebral metabolism, as well as levels of markers of brain injury in plasma and cerebral microdialysate fluid.

## Methods

### Animals

In all, 22 Norwegian landrace breed piglets of both sexes were included. No specific genetic strain of piglets was pursued or used. All animals, aged 9–12 weeks, were healthy and sexually immature. The animals were procured from a private farm, Mångsbo Gård, Uppsala, Sweden. On the morning of each experiment, two healthy animals, as assessed by the experienced provider, were separated from the other animals and transported in individual containers to the research facility.

### Ethical statement

The study was designed with consideration of Minimum Quality Threshold in Pre-clinical Sepsis Studies (MQTiPSS) [22] and reported in adherence to the Animal Research: Reporting of In Vivo Experiments (ARRIVE) guidelines [23]. The study was approved by the Animal Ethics Board (Uppsala Djurförsöksetiska Nämnd, permit no. C250/11) in Uppsala, Sweden. Handling of the animals was done according to the guidelines of the Swedish Board of Agriculture. All measures were taken to decrease suffering. The animals were free to eat and drink ad libitum up to 1 h (h) before the start of the experiment. All surgical procedures were performed under general anaesthesia and signs of pain were monitored and treated. Immediately after the experimental endpoint, the animals were euthanised by an intravenous (i.v.) injection of 10 mL potassium chloride. Following the subsequent asystole, mechanical ventilation was withdrawn.

### Anaesthesia

General i.v. anaesthesia was induced with zolazepam 3 mg × kg^−1^, tiletamine 3 mg × kg^−1^, xylazine 2.2 mg × kg^−1^ and atropine 0.04 mg × kg^−1^. A bolus i.v. injection of ketamine 100 mg and morphine 20 mg was administered before securing the airway by surgical tracheostomy. Thereafter, MV (Servo 900C or Servo I, Siemens Elema, Stockholm, Sweden) was initiated and continued until the end of the experiment. Anaesthesia was maintained by continuous i.v. infusion of sodium pentobarbital 8 mg × kg^−1^ × h^−1^ and morphine 0.26 mg × kg^−1^ × h^−1^ dissolved in a glucose solution of 2.5% concentration, resulting in a fluid administration rate of 15 ml × kg^−1^ × h^−1^. Rocuronium bromide 2 mg × kg^−1^ × h^−1^ was administered by a separate infusion.

### Surgery

After bilateral paratracheal skin incisions, the thyroglossal arteries and jugular veins were identified with blunt dissection. A branch of the right thyroglossal artery was cannulated using a 5F catheter to sample blood and monitor blood pressure. A central venous catheter and a 7F pulmonary artery catheter were then placed via the right external jugular vein. On the left side, the internal jugular vein was cannulated using a 5F arterial catheter advanced 5 cm cranially to approximate the jugular bulb. A flow meter was applied to the left internal carotid artery. Through a small laparotomy, the urinary bladder was catheterised. After placement of the pig in the left side position, in which the animal remained so throughout the experiment, preparatory neurosurgery was initiated. Following a frontal flap incision, stretching medially from between the eyes to between the ears of the pig, the skull was trepanned with care taken not to injure the dura mater. The total area of the trepanation covered approximately 2 cm^2^. The superior sagittal vein was cannulated with a 22 G catheter to collect blood samples. A Clarke electrode, LiCox (Mediplast AB) was introduced to the brain parenchyma in 50% of the animals for registration of the partial oxygen pressure in brain tissue (p_bt_O_2_). One catheter for registration of intracranial pressure (ICP) and one for microdialysis were also introduced to the brain parenchyma.

After preparatory surgery, an i.v. fluid bolus of Ringer’s acetate 20 ml × kg^−1^ was administered and a stabilisation time of 30 min (min) allowed, after which baseline physiological values were recorded, blood samples collected and the abdominal fascia and skin sutured.

### Protocol

**Fig. 6:**
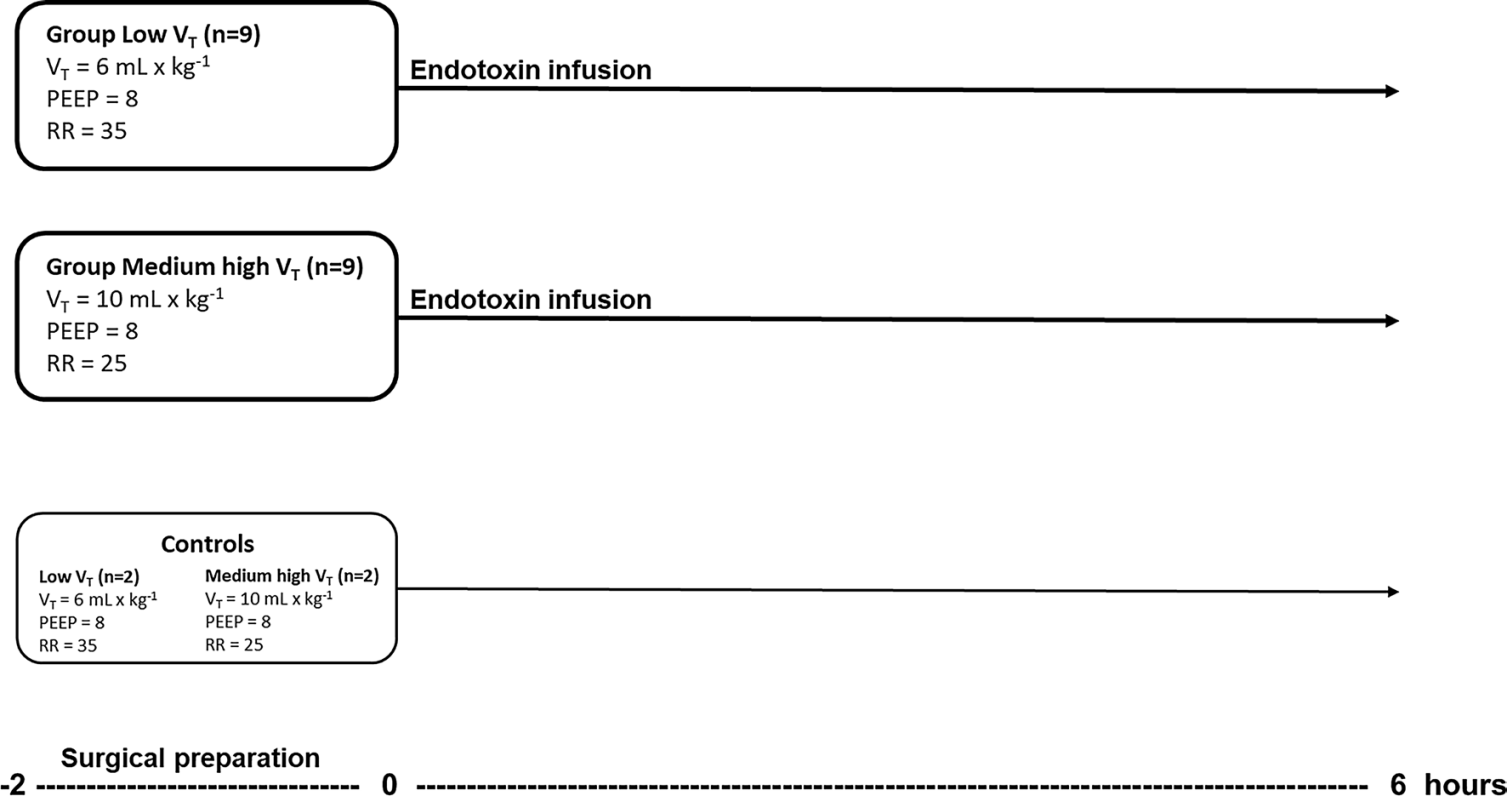
Design of the experiment

The animals were randomised to two experimental sepsis groups: Low V_T_ (n = 9) or Medium high V_T_ (n = 9), or two corresponding control groups not receiving systemic inflammatory-inducing endotoxin: Low V_T_ Control (n = 2) or Medium high V_T_ Control (n = 2). The design of the experiment is depicted in Fig. 6.

In the Low V_T_ and the Low V_T_ Control groups PEEP was set to 8 cmH_2_O, tidal volume (V_T_) to 6 mL × kg^−1^ and respiratory rate to 35 × min^−1^. Animals in the Medium high V_T_ and Medium high V_T_ Control group received the same PEEP, 8 cmH_2_O, but a V_T_ of 10 mL × kg^−1^ and a respiratory rate of 25 × min^−1^. Volume control mode, with an inspiratory to expiratory (I:E) ratio of 1:2 and a fraction of inspired oxygen (FiO_2_) of 0.3, was initially used in both groups. Immediately after preparatory surgery, a lung recruitment manoeuvre was performed by a stepwise increase of PEEP until an inspiratory plateau pressure of 30 cmH_2_O was reached. Then inspiratory pressure was kept constant for 10 s. An infusion of Ringer’s acetate, 20 mL × kg^−1^ was set for 30 min. During that period, ventilator adjustments were made to achieve normoventilation, i.e. an arterial partial pressure of carbon dioxide (P_a_CO_2_) between 5.0–5.5 kPa before the start of the experiment at 0 h.

At 0 h, an i.v. challenge of endotoxin was started at 0.25 µg × kg^−1^ × h^−1^ and kept for the remainder of the experiment.

### Interventions

A goal-directed intervention protocol was used for the experiment set-up to mimic an intensive care setting of postoperative sepsis. Adjustments of the FiO_2_ and respiratory rate (RR) were made on the ventilator during the experiment. FiO_2_ was tuned by increments or decrements of 0.1 to maintain arterial partial pressure of oxygen (PaO_2_) within 10 to 30 kPa. RR was increased or decreased by 10% to maintain PaCO_2_ between 4.5 and 6.5 kPa. After every adjustment of ventilation, an arterial blood gas analysis was performed to evaluate ventilatory changes.

Circulatory management during the experiment was based on infusion of crystalloid fluids and noradrenaline. If mean arterial pressure (MAP) approximated mean pulmonary arterial pressure (MPAP), a 1 mL i.v. bolus of noradrenalin 20 µg × mL^−1^ was administered and followed by a continuous i.v. infusion of noradrenaline 20 µg × mL^−1^ at a rate of 5 mL × h^−1^ and an i.v. fluid bolus of Ringer’s acetate 15 ml × kg^−1^ × h^−1^ rapidly infused. The fluid bolus was allowed to be repeated to a maximum additional fluid, outside the ordinary fluid protocol, to 30 ml × kg^−1^ × h^−1^.

Noradrenalin infusion was initiated if MAP values of less than 60 mmHg or cardiac index (CI) values of less than 2.0 L × min^−1^ × m^−2^ were recorded. When values of MAP exceeding 70 mmHg were recorded, the infusion of noradrenalin was decreased or discontinued.

Adjustments of the rate of infusion of noradrenalin 20 µg × mL^−1^ were made in the following steps: 0 ml × h^−1^ 5 ml × h^−1^ 10 ml × h^−1^20 ml × h^−1^40 ml × h^−1^. Combined paper/plastic covers were applied to the body of the animals to prevent hypothermia. The covers were removed if body temperature exceeded 42.5° C. No additional heating sources were applied.

### Analyses

Collection of physiologic data and samples from blood were done hourly, as were blood gas analyses performed on blood samples from the pulmonary artery, jugular bulb, thyroglossal artery and superior sagittal sinus. Samples from cerebral microdialysis were collected every second hour. The blood samples, in sodium heparin, were centrifuged to retain plasma and immediately frozen for later analyses. Samples from cerebral microdialysis were immediately frozen in cuvettes without additives. Commercial porcine-specific sandwich enzyme-linked immunosorbent assay (ELISA) was used to determine TNF-α and IL-6 in plasma (DY690B (TNF-α) and DY686 (IL-6), R&D Systems, Minneapolis, MN, USA and KSC0102 (IL-10), Invitrogen, Camarillo, CA, USA). The lower detection limits in sodium heparin plasma were 230 pg × mL^−1^ for TNF-α and 60 pg × mL^−1^ for IL-6. All ELISAs had intraassay coefficients of variation (CV) of < 5% and a total CV of < 10%. S100B was measured by CanAg S100 EIA assay (Fujirebio Diagnostics, Gothenburg, Sweden). The monoclonal antibodies in the enzyme-linked immunosorbent assay are raised against bovine S100B. CanAg S100 is a sandwich assay performed in microstrips coated with streptavidin. This S100B assay measures an equal amount of S100A1B and S100BB. The detection limit for S100B was 10 ng × L^−1^. The analytical precisions of S100 were 1.3–2.5% CV (intraassay) and 1.5–2.5% CV (interassay). Analyses of microdialysis samples were performed using kinetic enzymatic methodology and a single ray filter photometry detector. The instrument used was ISCUS^*flex*^ Microdialysis Analyzer (M Dialysis AB, Stockholm, Sweden) with a total CV of less than 10%. Lower level of detection for glucose was 0.02 mmol × L^−1^, lactate 0.02 mmol × L^−1^, pyruvate 2 µmol × L^−1^, glycerol 2 µmol × L^−1^ and glutamate 1 µmol × L^−1^.

### Calculations and statistics

Static pulmonary compliance was calculated during an inspiratory pause for 2 s when expired tidal volume divided by the difference between peak airway pressure and PEEP, were used. To calculate the net cerebral contribution of different biomarkers of metabolism and inflammation the product of the trans-cerebral concentration difference for each biomarker and the cerebral blood flow was calculated. The trans-cerebral concentration difference was calculated as the difference between the efferent plasma concentration, i.e. the superior sagittal sinus and the afferent plasma concentration, i.e. the artery.

The available amount of plasma available for analyses of S100B was not sufficient in sagittal sinus blood. Thus, this analysis was only performed in arterial and jugular bulb samples. For this reason, cerebral production of S100B was not calculated.

Based on previous studies, the power analysis was based on a detectable difference of 10% of TNF-α in systemic plasma during the experiment, an alpha error of 0.05, a power of 0.8 and an SD of 10%, yielding at least nine evaluable animals per group. All parameters approximated the normal distribution, except for the cytokines that were log-normally distributed. Urine output was non-normally distributed. For inference testing of group differences, a general linear model (GLM) with random effects was used. Random effects were introduced into the model to account for the within-subject dependencies of the time and the repeated measures [24]. The GLM equations were therefore a mix of fixed and random factors, i.e. mixed models. Statistica™ (Version 13.5, Statsoft, Tulsa, OK) was used in the statistical calculations and for the control of relevant assumptions.

## Supplementary Information

**Additional file 1**: **Table S1** Respiratory, circulatory and organ functions variables during the experiment.

## Data Availability

The dataset supporting the conclusions of this article is available for download for all registered Synapse users in the Synapse repository, [Synapse ID: syn22312989].

## Abbreviations

ARDS: Acute respiratory distress syndrome
ARRIVE: Animal Research: Reporting of In Vivo Experiments guidelines
CI: Cardiac index
CMRO_2_: Cerebral metabolic rate of oxygen
CPP: Cerebral perfusion pressure
CV: Coefficient of variation
ELISA: Enzyme-linked immunosorbent assay
F: French
F_i_O_2_: Fraction of inspired oxygen
G: Gauge
GLM: General linear model
ICP: Intracranial pressure
I:E: Inspiratory to expiratory ratio
IL-6: Interleukin 6
i.v.: Intravenous
MAP: Mean arterial pressure
MPAP: Mean pulmonary arterial pressure
Min: Minutes
MV: Mechanical ventilation
MQTiPSS: Minimum Quality Threshold in Pre-clinical Sepsis Studies
P_a_CO_2_: Arterial partial pressure of carbon dioxide
P_a_O_2_: Arterial partial pressure of oxygen
p_bt_O_2_: Partial oxygen pressure in brain tissue
PEEP: Positive end expiratory pressure
RR: Respiratory rate
SAE: Septis-associated encephalopathy
S_jv_O_2_: Jugular bulb oxygen saturation
TNF-α: Tumour necrosis factor alpha
V_T_: Tidal volume
