# Supplementary material for: Lung-protective ventilation increases cerebral metabolism and non-inflammatory brain injury in porcine experimental sepsis
Source: BMC Neurosci. 2021 Apr 29;22:31. doi: 10.1186/s12868-021-00629-0 (PMC8082058; doi:10.1186/s12868-021-00629-0)
Supplement: Supplementary file 1 — Additional file 1: Table S1 Respiratory, circulatory and organ functions variables during the experiment. [file 12868_2021_629_MOESM1_ESM.docx]

| **Supplementary table** |  |  |  |  |  |
| --- | --- | --- | --- | --- | --- |
|  | **Group** | **0** | **2** | **4** | **6** |
| **TVe** | **Low V_T_ Control** | 164±2 | 164±6 | 165±3 | 165±4 |
| (cmH_2_0) | **Medium high V_T_ Control** | 246±16 | 243±20 | 245±17 | 245±12 |
| **RR** | **Low V_T_ Control** | 49±3 | 49±3 | 49±3 | 49±3 |
| (min^-1^) | **Medium high V_T_ Control** | 25±1 | 23±4 | 23±4 | 23±4 |
| **P Peak** | **Low V_T_ Control** | 19±2 | 20±2 | 20±1 | 21±1 |
| (cmH_2_0) | **Medium high V_T_ Control** | 24±2 | 23±2 | 24±2 | 25±2 |
| **Compliance** | **Low V_T_ Control** | 24±5 | 24±6 | 24±5 | 21±4 |
| (mL x cmH_2_0^-1^) | **Medium high V_T_ Control** | 23±4 | 21±1 | 24±3 | 19±1 |
| **PaO_2_** | **Low V_T_ Control** | 17.4±0.4 | 16.2±0.6 | 15.4±0.8 | 13.7±0.3 |
| (kPa) | **Medium high V_T_ Control** | 18.9±2.1 | 17.1±0.2 | 16.7±0.4 | 16.8±1.0 |
| **PaCO_2_** | **Low V_T_ Control** | 5.5±0.1 | 5.4±0.1 | 5.5±0.2 | 5.9±0.5 |
| (kPa) | **Medium high V_T_ Control** | 5.0±0.4 | 5.5±0.6 | 5.5±0.9 | 5.5±0.8 |
| **PaO_2_/FiO_2_** | **Low V_T_ Control** | 434±8 | 404±16 | 385±21 | 320±7 |
| (mmHg) | **Medium high V_T_ Control** | 472±53 | 426±5 | 416±8 | 392±23 |
| **Temp** | **Low V_T_ Control** | 39.2±0.2 | 39.9±0.1 | 39.9±0.6 | 39,9±0.3 |
| (°) | **Medium high V_T_ Control** | 38.8±1.1 | 39.4±0.7 | 39.4±0.1 | 39.5±0.1 |
| **HR** | **Low V_T_ Control** | 103±2 | 107±4 | 103±4 | 101±2 |
| (min^-1^) | **Medium high V_T_ Control** | 118±6 | 120±4 | 122±12 | 124±12 |
| **MAP** | **Low V_T_ Control** | 80±7 | 84±12 | 74±6 | 74±4 |
| (mmHg) | **Medium high V_T_ Control** | 74±20 | 71±11 | 74±6 | 77±5 |
| **MPAP** | **Low V_T_ Control** | 19±2 | 23±6 | 22±2 | 21±1 |
| (mmHg) | **Medium high V_T_ Control** | 15±2 | 17±3 | 18±1 | 18±1 |
| **ICP*** | **Low V_T_ Control** | 11 | 16 | 20 | 12 |
| (mmHg) | **Medium high V_T_ Control** | 8 | 10 | 11 | 11 |
| **CI** | **Low V_T_ Control** | 3.7±0.5 | 3.1±0.3 | 3.4±0.2 | 3.4±0.2 |
| (L x min^-1^ x m^-2^) | **Medium high V_T_ Control** | 3.6±0.2 | 3.6±0.1 | 4.3±0.4 | 4.0±0.4 |
| **Lactate** | **Low V_T_ Control** | 0.8±0.1 | 0.8±0.1 | 0.8±0.1 | 0.7±0.1 |
| (mmol x L^-1^) | **Medium high V_T_ Control** | 1.3±0.1 | 0.8±0.1 | 0.7±0.1 | 0.7±0.1 |
| **Diuresis** | **Low V_T_ Control** | 105(70-140) | 108(95-120) | 130(120-140) | 290(120-460) |
| (mL x h^-1^) | **Medium high V_T_ Control** | 308(95-520) | 285(150-420) | 475(460-490) | 350(300-400) |
| **Cerebral blood flow** | **Low V_T_ Control** | 0.25±0.06 | 0.21±0.01 | 0.23±0.02 | 0.24±0.01 |
| (mmHg) | **Medium high V_T_ Control** | 0.25±0.03 | 0.28±0.04 | 0.29±0.02 | 0.30±0.1 |
| **Cerebral vascular resistance*** | **Low V_T_ Control** | 257 | 355 | 271 | 266 |
| (dyne x s x cm^-6^) | **Medium high V_T_ Control** | 303 | 270 | 213 | 212 |
| **Cerebral oxygen delivery*** | **Low V_T_ Control** | 44±18 | 40±16 | 39±9 | 40±15 |
| (mL O_2_ x min^-1^) | **Medium high V_T_ Control** | 37±10 | 32±3 | 28±3 | 37±9 |
| **CMRO_2_*** | **Low V_T_ Control** | 22±6 | 16±1 | 16±2 | 15±4 |
| (mL x min^-1^ x 100g^-1^) | **Medium high V_T_ Control** | 18±4 | 16±1 | 13±2 | 15±1 |
| **Cerebral glucose consumtion** | **Low V_T_ Control** | 0.21±0.16 | 0.18±0.06 | 0.18±0.05 | 0.19±0.10 |
| (mmol x h^-1^) | **Medium high V_T_ Control** | 0.19±0.07 | 0.15±0.04 | 0.14±0.11 | 0.13±0.02 |
| **Cerebral lactate production** | **Low V_T_ Control** | -0.01±0.04 | -0.01±0.03 | 0.01±0.01 | -0.01±0.01 |
| (mmol x h^-1^) | **Medium high V_T_ Control** | 0.04±0.01 | 0.02±0.02 | 0.02±0.02 | 0.01±0.02 |
| **Cerebral CO_2_-production** | **Low V_T_ Control** | 0.6±0.1 | 0.5±0.1 | 0.4±0.1 | 0.4±0.1 |
| (kPa x h^-1^) | **Medium high V_T_ Control** | 0.5±0.1 | 0.5±0.1 | 0.4±0.1 | 0.4±0.1 |
| **Log_10_ TNF-α** | **Low V_T_ Control** | 1.8±0.1 | 1.9±0.2 | 1.9±0.1 | 1.8±0.1 |
| (ng x L^-1^) | **Medium high V_T_ Control** | 1.7±0.4 | 1.9±0.2 | 1.7±0.2 | 1.8±0.1 |
| **Log_10_ IL-6** | **Low V_T_ Control** | 2.1±0.1 | 2.2±0.1 | 2.4±0.2 | 2.5±0.3 |
| (ng x L^-1^) | **Medium high V_T_ Control** | 2.0±0.1 | 2.0±0.1 | 2.1±0.2 | 2.1±0.1 |
| **S-100B Jugular vein*** | **Low V_T_ Control** | 0.9 | 9.3 | 6.1 | 10.8 |
| (ng x L^-1^) | **Medium high V_T_ Control** | 0.9 | 10.7 | 10.7 | 2.9 |

Results from the animals not exposed to endotoxin during the experiment. The animals in group Low V_T_ Control (n=2) were ventilated with a V_T_ of 6 mL x kg^-1^ while the animals in group Medium high V_T_ Control (n=2) were ventilated with a V_T_ of 10 mL x kg^-1^. Values are given av mean±SD for all variables except for diuresis, which is given av median (interquartile range). *denotes available data from only one animal, which prevents calculating SDs.

Abbreviations: TVe-expired tidal volume, RR-respiratory rate, P Peak-peak airway pressure, Compliance-Static airway compliance. PaO2-arterial partial pressure of oxygen, PaCO2-arterial partial pressure of carbon dioxide, PaO2/FiO2-arterial partial pressure of oxygen to fraction of inspired oxygen, Temp-core body temperature, HR-heart rate, MAP-mean arterial pressure, MPAP-mean pulmonary arterial pressure, ICP-intracranial pressure, CI-cardiac index, CMRO2- cerebral metabolic rate of oxygen, TNF-α- tumour necrosis factor alpha, IL-6- interleukin 6.
